# Machine learned features from density of states for accurate adsorption energy prediction

**DOI:** 10.1038/s41467-020-20342-6

**Published:** 2021-01-04

**Authors:** Victor Fung, Guoxiang Hu, P. Ganesh, Bobby G. Sumpter

**Affiliations:** 1 Center for Nanophase Materials Sciences, Oak Ridge National Laboratory, Oak Ridge, TN 37831 USA; 2 Department of Chemistry and Biochemistry, Queens College of the City University of New York, Queens, NY 11367 USA

**Keywords:** Catalysis, Computational chemistry, Materials for energy and catalysis

## Abstract

Materials databases generated by high-throughput computational screening, typically using density functional theory (DFT), have become valuable resources for discovering new heterogeneous catalysts, though the computational cost associated with generating them presents a crucial roadblock. Hence there is a significant demand for developing descriptors or features, in lieu of DFT, to accurately predict catalytic properties, such as adsorption energies. Here, we demonstrate an approach to predict energies using a convolutional neural network-based machine learning model to automatically obtain key features from the electronic density of states (DOS). The model, DOSnet, is evaluated for a diverse set of adsorbates and surfaces, yielding a mean absolute error on the order of 0.1 eV. In addition, DOSnet can provide physically meaningful predictions and insights by predicting responses to external perturbations to the electronic structure without additional DFT calculations, paving the way for the accelerated discovery of materials and catalysts by exploration of the electronic space.

## Abstract (web summary)

Computational catalysis would strongly benefit from general descriptors applicable for predicting adsorption energetics. Here the authors propose a machine-learning approach for adsorption energy predictions based on learning the relevant descriptors in a surface atom's density of states as part of the training.

## Introduction

Following the development of robust quantum chemistry software and the availability of high-performance computing, high-throughput screening is now an increasingly widespread approach for materials discovery^1–7^. The field of heterogeneous catalysis is no exception to this trend^8–11^. Individual studies in the range of 10^2^–10^3^ unique materials using quantum chemistry are now commonplace, and more extensive studies can reach up to 10^4^ candidates for properties such as adsorption energies on surfaces. The primary bottleneck here remains in the high computational cost of multiple geometry optimizations on extended surfaces, generally obtained using density functional theory (DFT). To bypass the need for DFT calculations, one such strategy has been to discover “descriptors” or “features” that can relate to the adsorption energy, often via linear scaling relations^12–14^. Ideally, the feature is chosen such that it is a more affordable quantity to obtain than the desired property to predict. These features generally come in three major categories: geometric, electronic, and energetic. Geometric features are those obtained from atomic positions, such as coordination numbers^15–18^, atomic symmetry functions^19–21^, and graph representations^22–24^. Electronic features are obtained from the electronic structure of a system, usually from a single-point DFT calculation. One of the most well-known examples of this is the d-band center in heterogeneous catalysis, which relates the position of the d-band in the electronic density of states (DOS) to the adsorption energy on transition metals^12,14,25^. In the last category are energetic features, which include adsorption energies, bulk formation energies, and vacancy formation energies obtained from geometry optimization. For example, the relationships between adsorption energy of hydrogenated adsorbates and their monatomic counterparts are well established via scaling relationships^12,26,27^

With these features in hand, properties such as adsorption energy can be predicted using a suitable regression model (Supplementary Table 1). In early studies, linear regression was most commonly used for its computational simplicity and physical interpretability. However, in recent years, more involved machine learning (ML) methods have become an appealing alternative to provide predictions especially where multiple features or non-linear relationships are involved^28–31^. These include Gaussian processes regression^32,33^, kernel ridge regression^34^, random forests^35^, and neural networks^19,36^. For geometric features such as connectivity graphs, convolutional neural networks (CNNs) have been used to predict various physical and chemical properties of bulk crystals, molecules and surfaces^22–24^. Compressed sensing methods such as LASSO and SISSO have also been used to select the most relevant features for regression^37–39^. Ultimately, the effectiveness of the ML model for prediction is still largely dependent on the quality or relevance of the initial features chosen for the input. Consequently, there is a considerable demand to engineer effective features that can be used in ML, especially ones which are applicable to a wide range of materials and environments.

For a given study, a choice must eventually be faced with regards to which class of features to use in ML. Geometric features such as coordination and connectivity are computationally trivial to evaluate and therefore affordable for high-throughput screening, but generally requires an expensive investment of 10^4^–10^5^ data entries to train^21–24^. Next, there are electronic features like the *d*-band center, which, requiring a DFT calculation, are more expensive to obtain. However, we find in general these features tend to need a smaller training set size to achieve a similar level of accuracy as the geometric features^36,38^. Finally, energetic features such as adsorption energy and vacancy formation energy are again more expensive to obtain than the previous class of features, but can be utilized with even smaller datasets on the order of ~10^1^–10^2^, especially in the case of linear scaling relationships^26,27,40–42^. We note that while there are certainly exceptions to this trend with respect to the number of training data required, this general order of features usually holds for problems of the same scale and complexity. This is likely due to structural features being more physically removed from the property being predicted (i.e., adsorption energy) than electronic features, and consequently require more training data to learn their relationships.

In this work, we focus on the electronic features for adsorption energy prediction, as it generally offers a good compromise between training and screening cost and requires a training data set size which is commonly obtainable in current high-throughput studies (~10^2^–10^3^ entries). It is well-known that the electronic structure of a surface is closely linked to its surface chemistry, indeed, many parallels to frontier molecular orbital theory have been made^43,44^. The electronic DOS of the surface, much like the orbitals in a molecule, directly determines the mode and strength of its interaction with adsorbates through the hybridization and formation of new orbitals/states. Electronic features such as the *d*-band center are derived from this basis, by ultimately reducing the overall DOS of the system to a single numerical quantity or feature which roughly correlates to the position of the resultant antibonding states formed from the surface-adsorbate interaction. However, despite its conceptual simplicity, the *d*-band center does not extend well across a diverse range of surfaces or adsorbates, leading to the development of additional features involving higher-order moments of the *d*-band, such as its width, skew and kurtosis^39,45,46^. In addition to the position and shape of the *d*-band, the number or filling of states, particularly near the Fermi level, have been found to be important quantities governing both repulsive and attractive interactions^38,39,47,48^. Unfortunately, the current situation in using electronic features is such that no single feature is applicable for all materials when screening for a diverse set of materials. Furthermore, these pre-existing features must also be discovered or selected prior to the study, which may not always be possible when moving to unexplored chemical spaces.

In this study, we explore a robust and more broadly applicable approach, where the electronic features are (intentionally) left for the ML model to discover. We aim to develop a general method to featurize the DOS for a wide range of materials with minimal human intervention or knowledge inserted into the framework, by using CNNs. These networks generally consist of convolution and pooling layers and are well-established for feature extraction in image recognition for two-dimensional data; they have also been applied to one-dimensional data^49,50^. Conceptually, one can view the (average) pooling layers in the neural network as functionally similar to the idea of quantifying the number or filling of states in the DOS for a particular energy range. Meanwhile, convolutional layers can be parameterized to recognize shapes and contours which can also be comparable to the goal of obtaining *d*-band moments such as skew or kurtosis. A major advantage here of using CNNs is in the dramatically greater flexibility in featurization which is not possible with pre-defined features, and which can furthermore be better tailored to the system or adsorbate being studied via training.

## Results

### Model architecture and training data

**Fig. 1:**
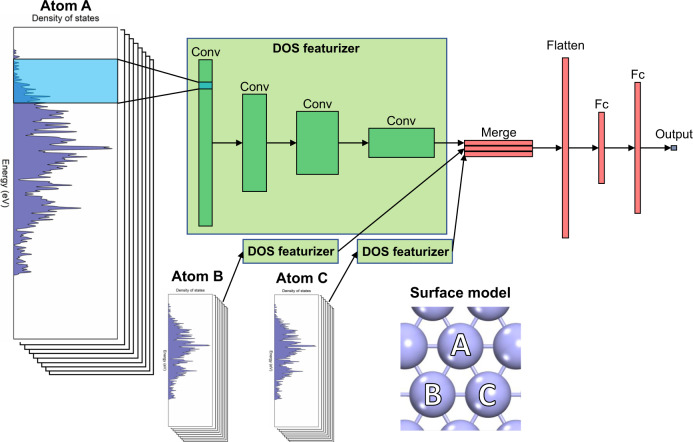
General schematic of the DOSnet model. The site-projected DOS of a surface atom serves as the input (light blue) which goes through a series of convolutional layers (green), followed by fully connected layers (red) and a final output layer. For additional atoms, the same convolutional layers are used with shared weights before being merged with the fully connected layers.

We develop a ML model, DOSnet, which takes the DOS directly as the input and extracts these features using CNNs as part of the training process (Fig. 1). The input of DOSnet is the site and orbital projected DOS of the relevant surface atoms participating in chemisorption, each comprising a separate channel. For example, the input for adsorption on the top site of a surface is the DOS of the one surface atom split into nine channels (s, p_y_, p_z_, p_x_, d_xy_, d_yz_, d_z2-r2_, d_xz_, d_x2–y2_). The input for adsorption on a bridge site will be the DOS of the two bridging surface atoms for a total of eighteen channels, and for the hollow site the input will contain a total of 27 channels, corresponding to the three nearest neighbor surface atoms. We include up to three atomic DOS as inputs for this dataset, but additional DOS inputs can be easily included as needed. The DOS input for each atom is then fed separately into a convolutional network (a DOS featurizer) with shared weights. The resolution of the DOS used in this work is 0.01 eV, though we find a similar performance even as a lower resolution is used, which can tuned in DOSNet by an initial average pooling layer (in this work the DOS is downsampled by a factor of 4). The data are further downsampled in the subsequent convolutional layers via strides and average pooling. After convolutions, the network is merged and flattened, and fed into the fully connected layer(s) prior to the output layer. Additional details of the network and hyperparameters can be found in the “Methods” section and SI (Supplementary Fig. 2).

To train our ML model, we used a dataset containing 37,000 adsorption energies on 2000 unique bimetallic alloy surfaces^51^. The bimetallic surfaces are comprised of 37 transition and non-transition metal elements in stoichiometric ratios of 0, 0.25, 0.5, 0.75, and 1. Adsorbates included in this set are the monatomic adsorbates H, C, N, O, S, and a selection of their hydrogenated counterparts, CH, CH_2_, CH_3_, NH, OH, SH. For each adsorbate, the adsorption energies have energy ranges of at least 2 eV to an energy range of up to 10 eV for C, N, and O which suggests an ample sampling of bonding ranging from very weak to very strong adsorption. The dataset used therefore represents a selection of important intermediates in catalysis adsorbed on surfaces exhibiting an extensive range of chemical environments. The DOS of these surfaces were not part of the published dataset and were computed separately in this work from the provided geometries. From computing just the *d*-band centers for the surfaces, it is evident they do not adequately follow the adsorption correlations described in the *d*-band theory across this set of materials (Supplementary Fig. 1), underscoring the need for a more robust and general predictive framework.

### Evaluation of ML performance

**Fig. 2:**
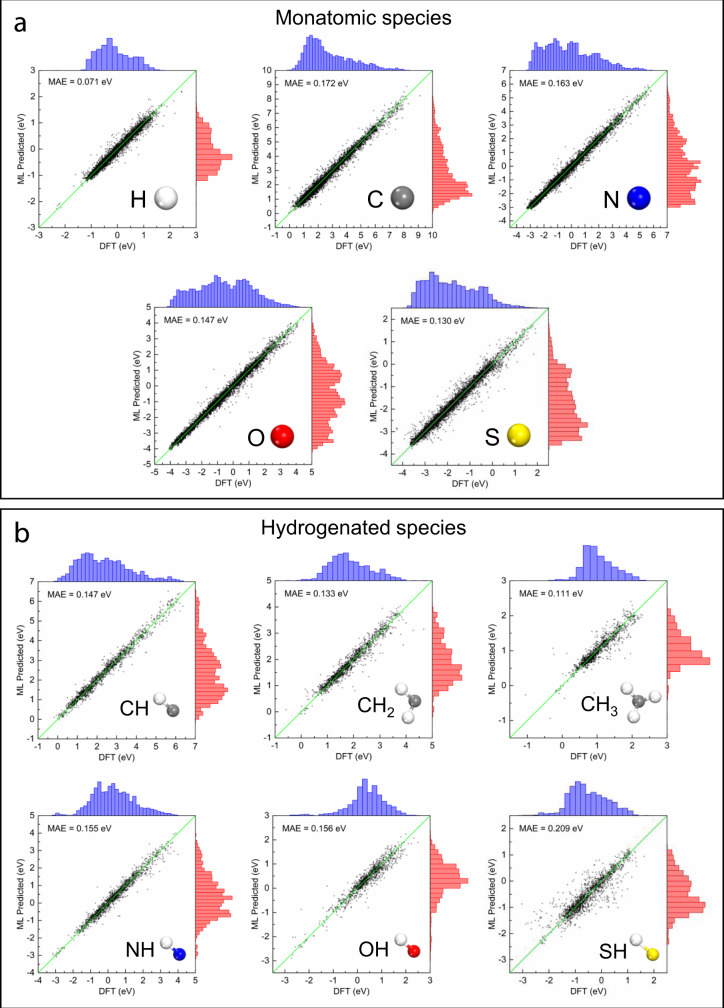
DOSnet performance for individual adsorbates. Parity plots and histograms between the DFT-calculated and DOSnet predicted energies from fivefold cross-validation are shown. Individual performances of DOSnet for each adsorbate are shown for **a** monatomic and **b** hydrogenated species.

We first test the performance of DOSnet for adsorption energy prediction for each individual adsorbate with the computed DOS as the input (Fig. 2). We used the same hyperparameters across all the adsorbates. From fivefold cross-validation, the weighted average of the MAE across the adsorbates is 0.138 eV. Of the monatomic adsorbates, H had the smallest MAE at 0.071 eV. Of the hydrogenated adsorbates, the MAE is fairly similar except for SH at 0.209 eV. We next compare the performance of DOSnet to the current state of the art approaches on data with similar diversity and breadth in materials composition and adsorption energies. Compared to a Gaussian process regression method trained on the same dataset used in this work and using 2-D connectivity, atomic properties, and *d*-band descriptors as features, DOSnet has a 0.06 eV lower MAE on average^32^. In the same work, when additional energetic information (adsorption energies of other adsorbates in the dataset) are incorporated through residual learning as features for the Gaussian process regression, it has a similar average MAE. Meanwhile, DOSnet is also competitive with respect to a state of the art graph-based ML approach, which has a MAE of ~0.12–0.15 eV on a separate dataset containing ~12,000 H adsorption energies on alloys^24^. Overall, DOSnet performs very well given it uses only DOS as input, without additional commonly used atomic features such as electronegativity, ionization potential, and atomic radius nor using the information on geometry and connectivity. These fitting results suggest the raw DOS of just the metal sites on the surface serve as a suitable input in generating an ‘electronic fingerprint’ of the surface system via DOSnet with a very competitive performance.

**Fig. 3:**
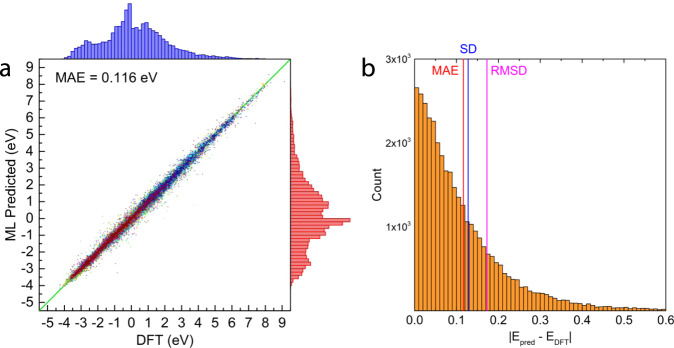
DOSnet performance on the combined dataset. **a** Parity plot and histogram between the DFT-calculated and DOSnet predicted energies on all 35,000 entries from fivefold cross-validation. **b** Histogram of prediction residuals.

Furthermore, we show it is possible to train a single instance of DOSnet across all adsorbates simultaneously by including an additional input representing the adsorbate (Supplementary Fig. 3). Currently, we simply use the DOS of the binding atom in the adsorbate (e.g., the C atom in CH_3_) in the gas phase. The weights of the convolutional layers responsible for featurizing the DOS are shared across the different adsorbate data sets. In doing so, the average MAE is further lowered by ~17% to be 0.116 eV from fivefold cross validation (Fig. 3). The error distribution for DOSnet shows a sharp unimodal distribution with a low population of outliers and a standard deviation of 0.127 eV. The root mean squared deviation (RMSD) is slightly higher than the MAE at 0.173 eV. The improved performance of this combined framework for DOSnet is possible due to transfer learning since chemisorption across the adsorbates is generally correlated to many of the same surface features. This approach is appealing as data from existing materials databases of adsorption energies can be leveraged to more efficiently train for additional adsorbates and reducing the amount of training data needed.

**Fig. 4:**
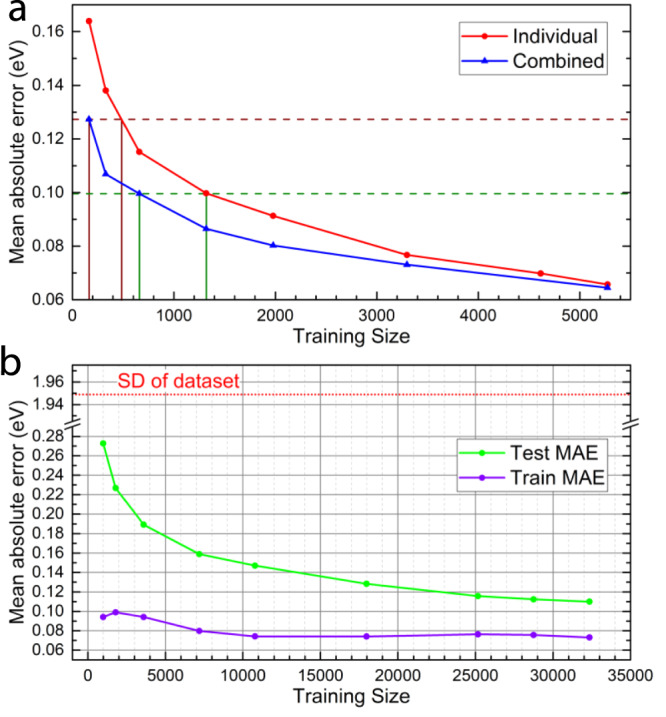
Training size dependence of DOSnet performance. **a** Mean absolute error versus training size for DOSnet on the hydrogen adsorbate. A comparison is made between DOSnet trained individually on H (red) and DOSnet trained while including all other adsorbates in the combined approach (blue). Colored guide lines highlight the reduced training data needed in the combined case which takes advantage of transfer learning. **b** Mean absolute error of the test set and train set versus training size for the combined DOSnet approach trained on all adsorbates.

We look at the prediction accuracy of DOSnet with respect to training size (Fig. 4), which provides an important estimate of the training data needed for a high-throughput screening application. First, we show that by training DOSnet across all adsorbates, the amount of training data needed can be reduced significantly by a factor of 2 or more via transfer learning. For example, in the case of H adsorption (Fig. 4a), to achieve a MAE of ~0.10 eV, ~1320 training data are needed when trained individually, but this is reduced to 660 data when trained simultaneously with the other adsorbates. The improvement is increased further for smaller training sizes, where 480 training data (individual) vs 164 data (combined) is needed to achieve the same MAE of ~0.13 eV. To reach a similar level of accuracy with graph neural networks with geometric features, ~12,000 training data are needed by comparison^24^. With regards to overall training size dependence, we find the testing error appears to converge to a MAE of 0.1 eV with around 30,000 training data, or 2700 training data per adsorbate (Fig. 4b). On the other end of the spectrum, even relatively small datasets can provide a reasonable accuracy. With 1800 data or around only 160 data per adsorbate, DOSnet can still provide a MAE of 0.23 eV, which is still well below the standard deviation of the dataset at 1.95 eV and suitable for search space reduction purposes^8^.

We also investigate the impact of surface optimization for our DOSnet training. Conventionally, surfaces are first optimized before obtaining and applying electronic descriptors for property prediction. In keeping with this convention and to provide a valid comparison of performance with the literature we also used the relaxed slabs for the DOS calculations so far. However, this presents a serious limitation in high-throughput screening applications because of the high computational cost of the geometry optimization step. Incidentally, this also defeats the purpose of using electronic descriptors over energetic descriptors if the goal is to use a feature with lower computational complexity. To test whether DOSnet can perform similarly well without requiring geometry optimization, we re-calculate the DOS for the unoptimized surfaces and train DOSnet with the new inputs (Supplementary Fig. 4). The performance is reduced marginally by 7.1% to a MAE of 0.125 eV (Table 1). Because DOSnet can be applied to the DOS from unrelaxed surfaces without a significant degradation in performance, this approach remains viable as a means of computational cost savings.

**Table 1:** Comparison of performance on relaxed and unrelaxed surfaces.

	Optimized	Unoptimized	Difference (%)
MAE (eV)	0.116	0.125	7.100
RMSD (eV)	0.173	0.190	9.481
*R*^2^	0.992	0.991	0.170

The upper-limit convergence of MAE at ~0.1 eV also provides a rough estimate on the limit of performance from using only the DOS as the input. Since several aspects of the surface-adsorbate interaction such as electrostatic interactions, charge transfer, and surface rearrangement are not well represented from the pre-adsorption DOS it should not be possible to achieve perfect parity; in that regard a MAE of 0.1 eV is already quite noteworthy. The lack of information on these additional interactions may also explain the comparatively poorer fitting for certain adsorbates and not others such as SH. To achieve better performance in future models, additional information that relates to these aspects of surface adsorption may be included in the ML model, either as secondary inputs or as separate networks in an ensemble scheme.

### Analysis of ML features and physical insights

**Fig. 5:**
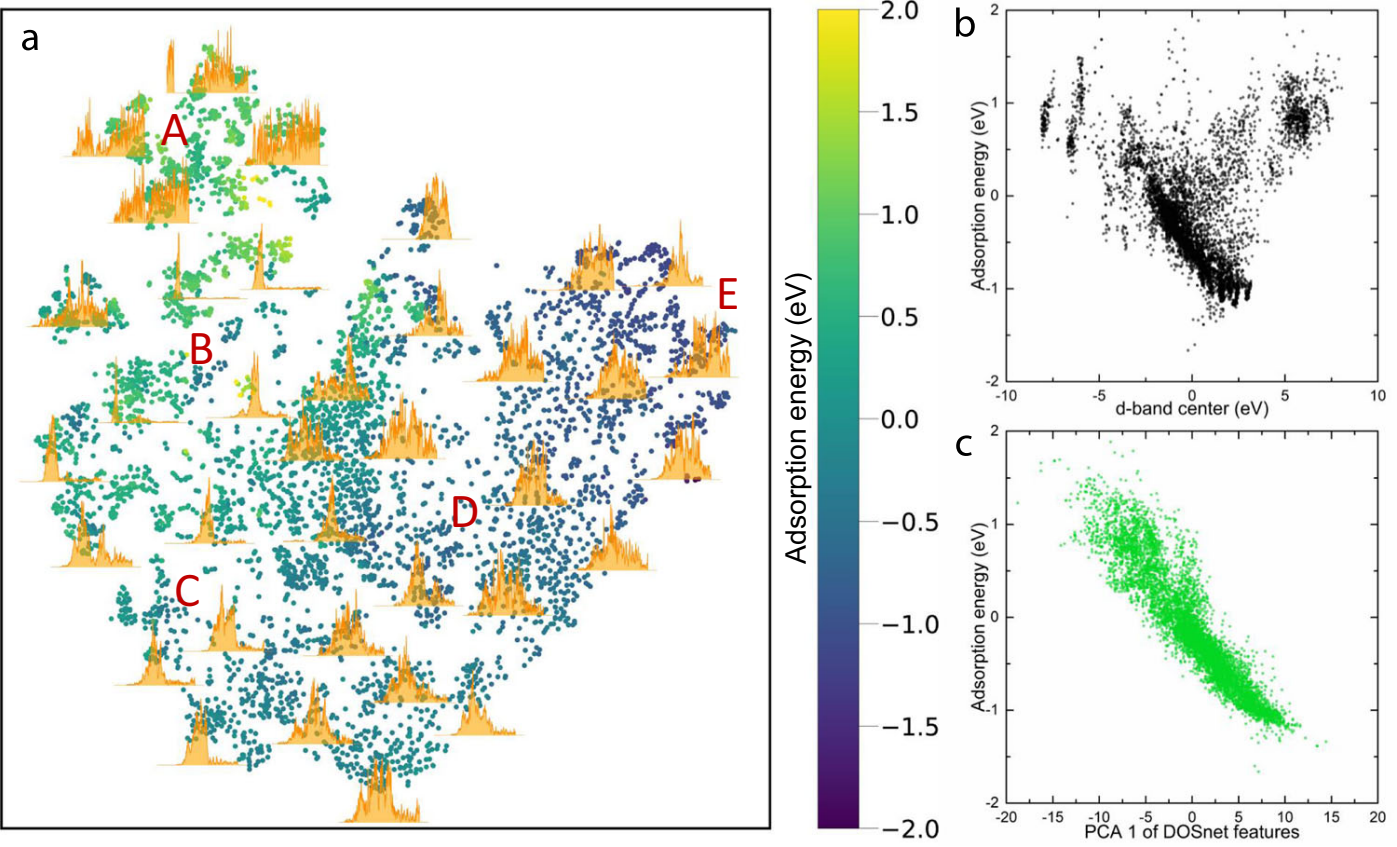
Visualization of DOS feature space and correlation to adsorption energy. **a** t-SNE plot of DOS features obtained from the last convolutional layer of DOSnet for the case of hydrogen adsorption. Points represent unique surface sites and are colored by the adsorption energy of that site. Selected DOS from these sites are plotted. **b** Plot of *d*-band center versus hydrogen adsorption energy. **c** Plot of the first component of the DOS features from a principal component analysis versus hydrogen adsorption energy.

Based on the excellent prediction performance of DOSnet, we believe the ML model has successfully obtained the relevant features in the electronic structure relevant to adsorption energy. To show this, we extract the output of the last convolutional layer of DOSnet, and use t-SNE, a well-established technique for dimensionality reduction and feature visualization^52^, to map them (using the H adsorbate dataset as an example; additional details in the methods section). The t-SNE plot in Fig. 5a corresponds closely to adsorption energy, going from regions of weak adsorption on the left to strong adsorption on the right in a generally continuous manner. Visual similarities within the regions can be easily seen, having been successfully captured by the ML features. Region A, with the weakest adsorption energies, are primarily comprised of alloys of non-transition metals (TMs) such as Ga, In, and Tl and with some late-TMs such as Au. Region B comprises of alloys of group 12 TMs which share pronounced peaks low in energy corresponding to completely filled d-states. Region C comprises of alloys of group 8–11 noble TMs such as Os, Pd, and Pt. Region D is predominantly mid to early-TM alloys, and region E contains the group 3–5 early TMs with many states in the high energy regions, and which have the strongest adsorption. This demonstrates our ML approach provides a physically meaningful latent space from which further explorations on electronic structure-property relationships can be explored, as well as inverse design applications that have only been applied to structural and compositional latent spaces so far^53^.

By obtaining the first principal component of the same set of features, we also find a much stronger correlation with hydrogen adsorption energy (*R*^2^ = 0.86) compared to the *d*-band center (Fig. 5b-c). In addition, because these features are drawn from all orbital contributions, surfaces containing non-TMs without a *d*-band can also be included in the same plot which are otherwise left out when utilizing the *d*-band center. The correlation is reasonably remarkably good provided only a single feature is used (PCA 1) and confirms that our approach can obtain highly effective features which can also be straightforwardly used in other ML regression methods. Naturally, the fitting here is worse than when we use the full DOSnet model since we only use the first principal component and do not account for the non-linear relationships between certain features and adsorption energy. This was captured in the full DOSnet model where additional fully connected layers were included after the convolutional ones.

To obtain further insights into the model, one can adopt a perturbative approach on the ML inputs by applying transformations on the input DOS and observing its effect on the ML prediction. This is particularly important, because in ab initio methods, it is impossible to probe the effects of such a transformation and interrogate how predictions might change without repeating an expensive solution to the Schrödinger equation. Furthermore, such transformations often cannot be applied directly. Here, we can apply transformations such as band-shifts, bandwidth-changes or suppression of certain states to the input DOS, corresponding to electron/hole doping, strain or alloying to the material and predict the effects on a property (such as adsorption energy) readily using DOSnet.

**Fig. 6:**
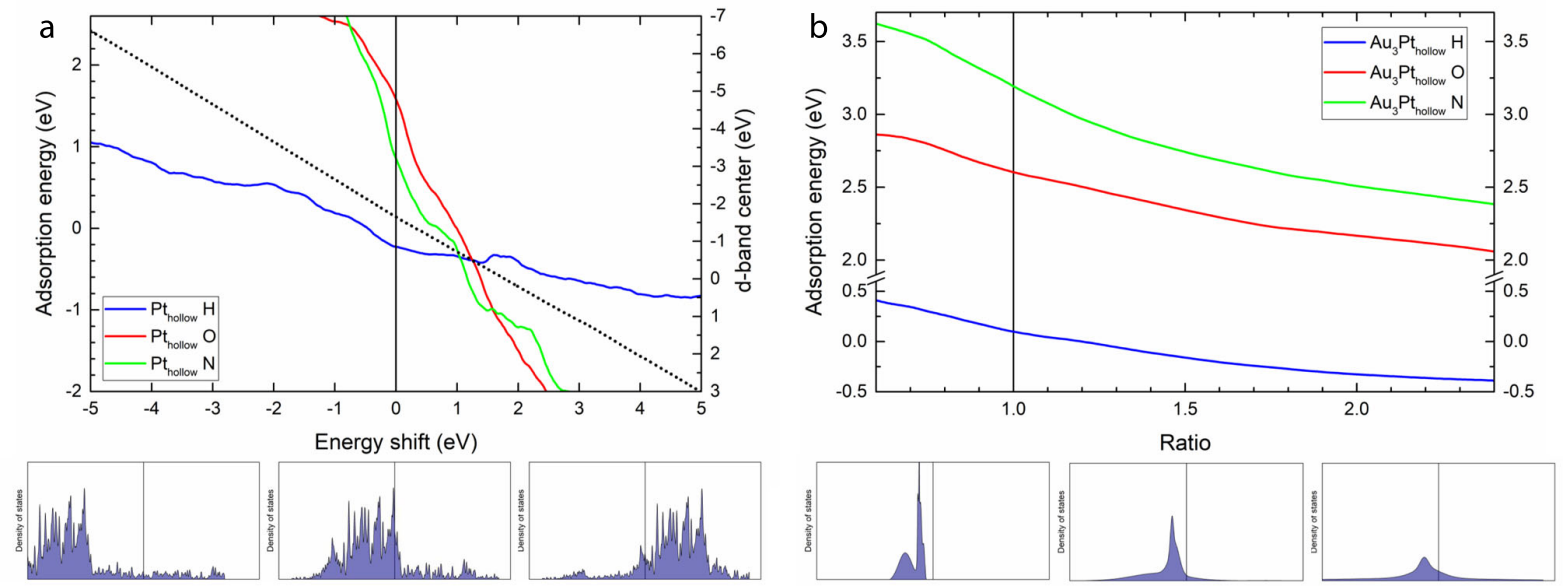
Effect of *d*-band changes on predicted adsorption energy. **a** Effect of *d*-band position on adsorption energy for the case of the Pt hollow site. **b** Effect of *d*-band width on adsorption energy for the case of the Au_3_Pt top site.

The DOS of a particular surface can be shifted along the energy axis, and the change in adsorption energy can be tracked as shown in Fig. 6a for the case of Pt. Shifting of energy in the DOS can occur, for example, in electron/hole-doping. The largely smooth and continuous plots in the ML adsorption energies as a function of the magnitude of the shift suggests a well-behaved model with an ample sampling of DOS in a region spanning roughly 10 eV in their *d*-band centers. For all studied adsorbates, a downshift of the Pt DOS leads to an increase in adsorption energy and vice versa, differing only by the slopes; we find this to be the case for other surfaces as well. If we track the *d*-band center, we see these are directly correlated, which is line with chemical intuition and the *d*-band theory. These results demonstrate that for a given distribution of the DOS, the position of the states taking part in bonding with the adsorbates, relative to the Fermi level, is linearly correlated to the adsorption energy. Next, we explore what happens when the distribution or shape of the DOS changes while the *d*-band center is constant.

We apply another transformation to the DOS by tuning of the *d*-band width, a similarly important factor in governing adsorption strength like the d-band center, which can be experimentally controlled by external strain. Here, we can approximate the DOS as a set of gaussian functions, whose width can be varied without changing the mean or area (representing the *d*-band center or a total number of states in the DOS). This allows us to independently investigate the effect of the *d*-band width on adsorption energy. We demonstrate this for the case of a Au_3_Pt alloy with a moderately localized DOS in Fig. 6b. Interestingly, we find a broader *d*-band width leads to a lower adsorption energy. This appears to run counter to the expectation in the literature where a narrower *d*-band leads to a lower adsorption energy. However, in previous theoretical studies, the *d*-band width is not an independent parameter due to its direct correlation with the *d*-band center, usually in the context of applying a tensile or compressive strain to a surface^54,55^. Consequently, when the *d*-band width is decreased in a surface, the *d*-band centers are also shifted upwards, making it difficult to deconvolute the separate contributions. Our study is not constrained by these limitations. One way to rationalize our observations is noting the fact that a broader surface *d*-band pushes more states to higher energies which also leads to higher energy surface-adsorbate antibonding states and therefore stronger bonding. While this analysis is by no means conclusive, we show that the freedom to apply transformation to ML inputs allows us to explore these different scenarios on adsorption energies, and provide guidance for further investigation using first-principles calculations or experiments.

**Fig. 7:**
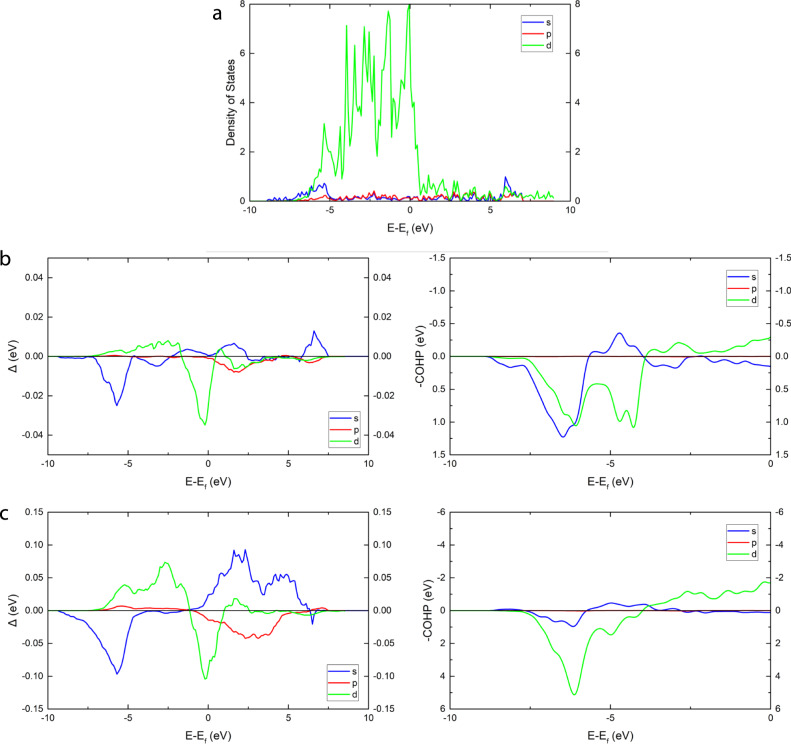
Occlusion sensitivity for predicted adsorption energy. **a** Density of states of the Pt hollow site. Occlusion sensitivity Δ, for **b** hydrogen adsorption and **c** oxygen adsorption on the Pt hollow site and crystal orbital Hamilton population (COHP) curves for the corresponding adsorbate surface interaction.

Finally, one can obtain insights into which parts of the DOS are responsible for the overall ML prediction by masking portions of the DOS input before making the prediction, which is sometimes referred to as an occlusion sensitivity analysis. Here, we apply a mask where all states are set to zero and move it along the *x*-axis in Fig. 7. The *y*-axis, Δ, is defined as the adsorption energy of the perturbed DOS minus the unperturbed DOS. A negative value means removing these states makes the adsorption energy more negative, suggesting that these states are responsible for the bonding interaction, and vice versa. Starting with hydrogen adsorption on Pt in Fig. 7b, we find positive values of Δ at low energies and negative values of Δ at higher energies for the *d* orbital contribution. Again, this is largely in line with chemical intuition where surface *d* states which are lower in energy contribute less to bonding, due to the lower position and greater filling of the resultant surface-adsorbate antibonding states. Calculation of the crystal orbital Hamilton populations (COHP) from DFT, which is more grounded on rigorous quantum-mechanics, yields a remarkably similar picture where the surface-adsorbate interaction is primarily bonding, but with a noticeable antibonding contribution near the Fermi level. The *s*-orbital contribution is primarily bonding from both occlusion sensitivity and COHP. However, when moving from hydrogen adsorption to oxygen adsorption, a much larger proportion of states have positive Δ, indicating a weaker net interaction (Fig. 7c). When compared with the COHP, we also find a much larger proportion of occupied antibonding states, consistent with the occlusion sensitivity analysis. Similarly, the COHP picture shows the *s* states are largely non interacting, and the occlusion sensitivity analysis shows roughly equal positive and negative contributions to adsorption energy.

These analyses suggest the DOSnet model has successfully learned key aspects of the relationship between the DOS and the surface-adsorbate interaction to provide accurate predictions of surface chemistry. While it is dangerous to take ML-based analyses at face value, these observations can be used to study hypothetical responses to external perturbations, such as doping, alloying, and strain, without any additional DFT calculations and raise important questions which can then be investigated with more rigorous methods. While we limit ourselves to just these simple transformations, DOSnet allows us to obtain the mapping $P\left( {O\left( x \right)} \right)$ for any property *P* to which the model was trained, when the underlying material characteristic (in this case, the DOS) *x* is transformed under an operation $O(x)$ in response to external perturbations with practically no additional cost. The addition of physical constraints to the ML model can potentially make more robust physical interpretations of these results, going beyond using ML simply as a black-box method.

### Computational savings and relevance to high-throughput studies

Screening with DOSnet is best leveraged on surfaces with a large quantity of unique adsorption sites, since only one single-point calculation is needed per surface. This makes the method suitable for studying bimetallic or multi-metallic surfaces such as high-entropy alloys or surfaces containing a multitude of different step or defect sites. It is also most efficient for studies with large sets of adsorbates, for example for catalytic reactions with complex reaction networks and many intermediates such as CO_2_ hydrogenation or alkane combustion. Potentially, the DOS of a surface may not even require a DFT calculation, but can be predicted by a suitable ML method as well, making the cost for screening negligible^56–58^. Nonetheless, a sizeable amount of training data containing adsorption energies are still needed prior to screening, as shown in Fig. 4. This can make small-scale predictions difficult to do compared to using conventional linear scaling relationships which can be fitted with just tens of data points. A possible solution to this problem is to start training from an existing model with weights pre-trained on a much larger benchmark dataset, taking advantage of transfer learning to reduce the data needed. The DOSnet model and weights from training on the aforementioned 37,000 adsorption energies have been made available for this purpose. Additional improvements such as applying physical constraints to the model and methods for dealing with multi-fidelity data can make this approach more widely applicable and will be the topic of future investigation. As computational databases continue to expand, including a recent example with ~500,000 adsorption energies^59^, the effectiveness of using pre-trained models will also likely improve significantly when working with limited data.

Finally, this work demonstrates the potential usefulness of compiling the DOS of surfaces to serve as an important component in materials databases. Prior to the development of a means of using the DOS directly for prediction, this information is of limited use for high-throughput screening except for providing fixed features such as band gap and the d-band center. However, with DOSnet, or with any other model which can featurize the DOS, it can provide a much more broadly applicable and information-rich source of data. Whereas other electronic information of the system such as charge density could also be useful, their prohibitively large file sizes make it impractical to store compared to the DOS. While databases containing DOS information are currently available for bulk crystal structures^60,61^, we note no comparable ones currently exist for surfaces and other solids.

## Discussion

We developed an ML model which takes the DOS of the surface atoms as input to provide accurate adsorption energy predictions (average MAE = 0.138 eV). A key aspect of this model is the use of one-dimensional convolutional filters which extracts features from the DOS relevant to adsorption and avoids the limitations in current pre-defined electronic descriptors. Including the DOS of the adsorbate allows the model to predict adsorption energies of an arbitrary adsorbate in a unified manner and provides better performance via transfer learning (MAE = 0.116 eV). This combined approach allows related adsorption data to be leveraged via transfer learning to reduce the training data needed. From the training size dependence of DOSnet, one can conclude it is best suited for applications where a moderate amount of training data is available (10^2^–10^3^ data entries per adsorbate). Because predictions can be made on unoptimized surfaces with only a minor decrease in accuracy (MAE = 0.125 eV), this approach can offer 2–3 orders of magnitude or more in computational cost savings over purely DFT. DOSnet therefore provides cost-effective, accurate energetic predictions with minimal human preparation or knowledge of existing features, making it suitable for high-throughput screening applications. A further analysis of the DOSnet features with **t**-distributed stochastic network embedding (t-SNE) shows it can distinguish from a wide diversity of DOS configurations and maps closely to the adsorption energy from PCA. We applied a number of transformations to the inputs, to interrogate effects of external perturbations such as doping, alloying or straining, by altering DOS position, width, and by selective masking of specific states. From these investigations we find DOSnet provides remarkable physical interpretability and insights to how adsorption energies would change under these perturbations. These examples demonstrate the potential benefits in using DOS-based ML methods to map out and obtain insights in the electronic structure chemical space, as well as accelerate predictions.

## Methods

The 37,000 adsorption energies were obtained from DFT calculations, with further details including force convergence, k-point sampling and energy cutoffs on the computational method described in the reference^51^. The DOS on the ~2000 surfaces were not included in the public repository, and were separately calculated in this work. These calculations were performed using the Vienna ab initio Simulation Package (VASP)^62,63^. The Perdew-Burke-Ernzerhof (PBE)^64^ functional form of generalized-gradient approximation (GGA) for electron exchange and correlation energies were used. While the original reference used the BEEF-vdW functional for the adsorption energy calculations, we found no significant difference in accuracy when using PBE for the electronic structure during the ML training. All calculations were performed with spin polarization. The projector-augmented wave method was used to describe the electron-core interaction^62,65^ with a kinetic energy cutoff of 450 eV for the surface calculations. A 6 × 6 × 1 sampling of Brillouin zone using a Monkhorst-Pack scheme was used for the k-points^66^. A Gaussian smearing of 0.05 eV was used for the DOS. The DOS in the range of −14–8 eV were used, with a resolution of 0.01 eV, for the ML portion.

The ML model was created and trained using the Keras library and sci-kit learn^67^ was used for data processing and cross validation. The input data were standardized by shifting the mean to zero and scaling to a variance of one over all channels. Unless otherwise noted, rectified linear unit activation functions were used in the convolutional and fully connected layers. A diagram of the model architecture and hypermeters can be found in Supplementary Figs. 1 and 2 for the DOSnet for individual adsorbates and all adsorbates, respectively. The total number of trainable parameters in the two DOSnet models are respectively 1,718,401 and 1,993,601. The Adam optimizer was used for training with an initial learning rate of 0.001 for a total of 60 epochs and a batch size of 16–128 depending on the system. A Logcosh loss function was used for its robustness to outliers that may be encountered in high-throughput databases. Training the DOSnet requires ~10–60 min on a single modern CPU; evaluating ~35,000 predictions on a trained model requires ~30 s.

The principal component and t-SNE analyses were performed using the sci-kit learn python package. The output of the last convolutional layer containing 3 × 150 filters is obtained, which is reduced to 100 with PCA to reduce noise. A perplexity of 50 was used for t-SNE. For the perturbative analyses, five identical but separately trained models were used with their outputs averaged to reduce noise. For the occlusion sensitivity analysis, a window size of 1 eV was used, with a stride of 0.1 eV. This was performed for each separate channel representing the orbitals; the p and d contributions were then averaged over the number of orbitals. Crystal orbital Hamilton populations were computed using LOBSTER from the DFT wavefunctions ^68^.

## Supplementary information

Supplementary Information

## Data Availability

The raw data used in this work are available from the corresponding authors upon reasonable request. Processed data for a subset of adsorbates are also included at https://github.com/vxfung/DOSnet.

## Code availability

The machine learning code used in this work is available at https://github.com/vxfung/DOSnet.
