# Supplementary material for: Machine learned features from density of states for accurate adsorption energy prediction
Source: Nat Commun. 2021 Jan 4;12:88. doi: 10.1038/s41467-020-20342-6 (PMC7782579; doi:10.1038/s41467-020-20342-6)
Supplement: Supplementary file 1 — Supplementary Information [file 41467_2020_20342_MOESM1_ESM.pdf]

Supplementary Information for

Machine Learned Features from Density of States for Accurate Adsorption Energy Prediction

Victor Fung,[1]* Guoxiang Hu,[2] P. Ganesh,[1] Bobby G. Sumpter[1]

[1]*Center for Nanophase Materials Sciences, Oak Ridge National Laboratory, Oak Ridge, Tennessee 37831, United States*

[2]*Department of Chemistry and Biochemistry, Queens College of the City University of New York, Queens, NY 11367, United States*

**E-mail: fungv@ornl.gov Tel.: +1-951-384-5242*

Supplementary Figures

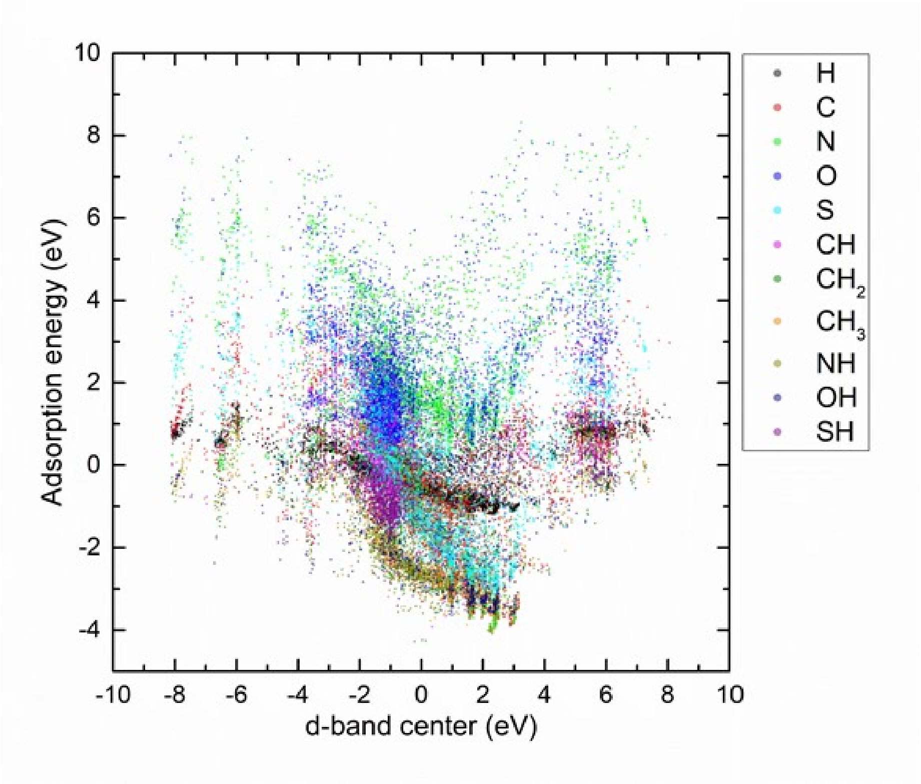

Supplementary Figure 1: *d*-band center versus adsorption energy. Correlation between *d*-band center and adsorption energy are shown for the different adsorbates, with surfaces with no *d*-bands excluded from the plot.

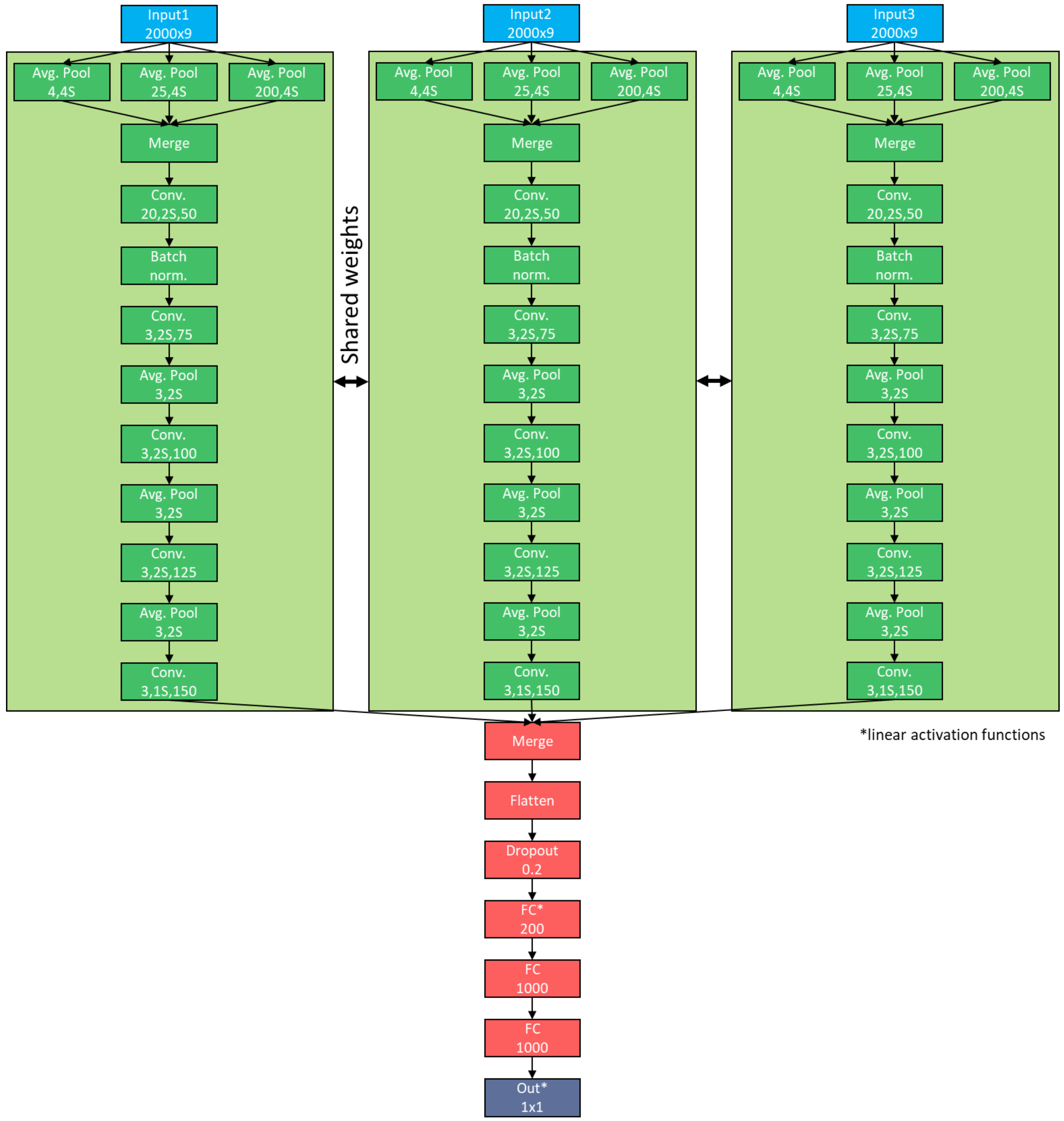

Supplementary Figure 2: DOSnet model architecture and hyperparameters for adsorbate-specific training. Avg. Pool is average pooling (numbers below are kernel size, stride), Conv. is 1D convolutional layer (numbers below are kernel size, stride, and filters), FC is fully connected layer (number below is number of neurons), Dropout is dropout layer (number below is dropout rate). All Conv. and FC layers use ReLU activation unless otherwise noted.

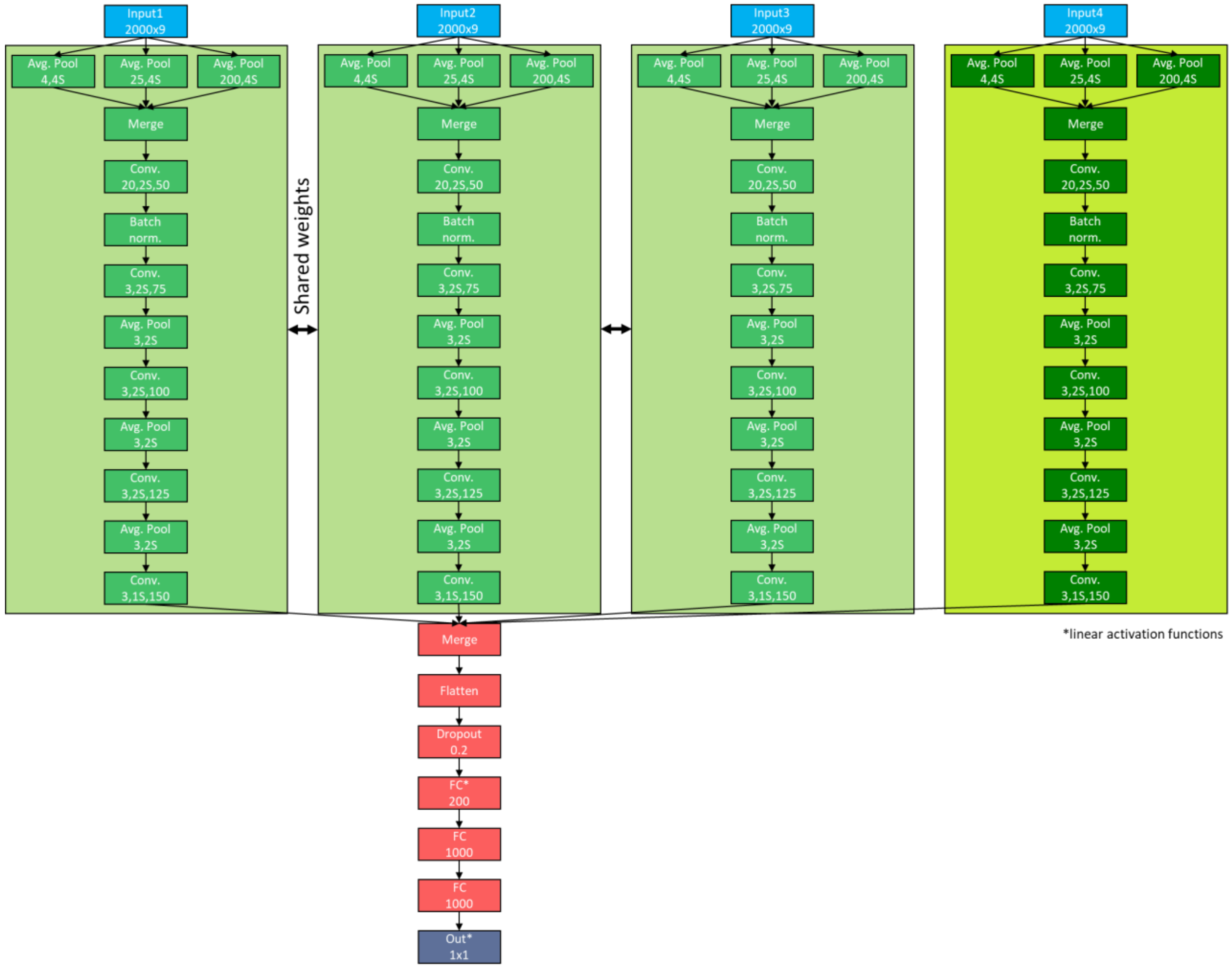

Supplementary Figure 3: DOSnet model architecture and hyperparameters for combined training. Avg. Pool is average pooling (numbers below are kernel size, stride), Conv. is 1D convolutional layer (numbers below are kernel size, stride, and filters), FC is fully connected layer (number below is number of neurons), Dropout is dropout layer (number below is dropout rate). All Conv. and FC layers use ReLU activation unless otherwise noted.

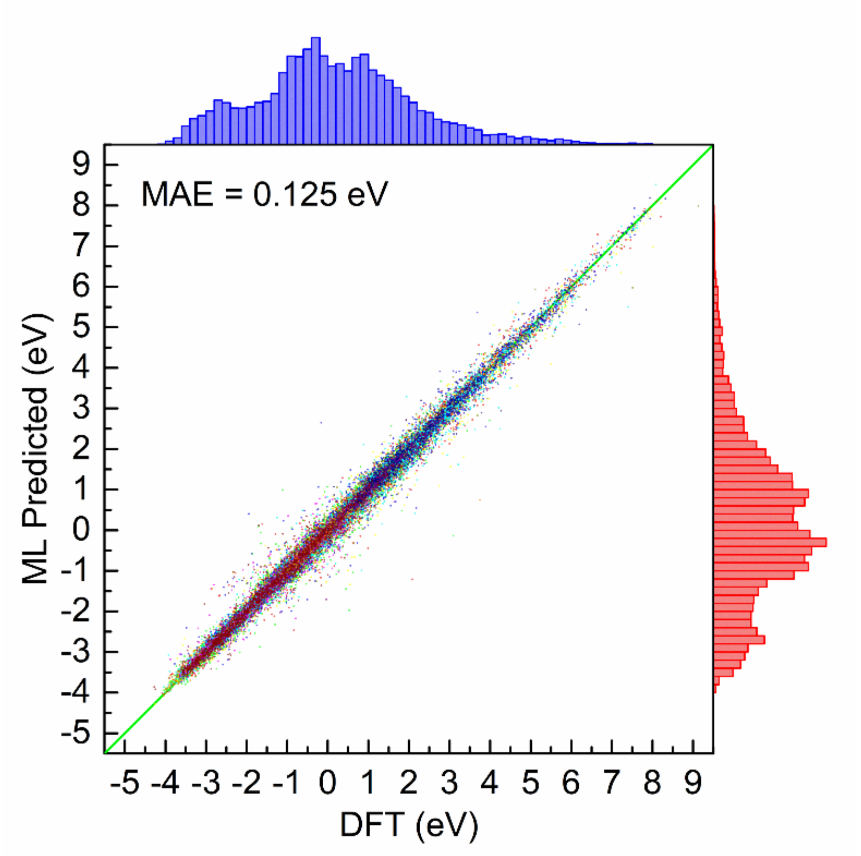

Supplementary Figure 4: DOSnet performance on the unrelaxed surfaces. Parity plot and histogram between DFT-calculated and DOSnet predicted energies are shown. Predicted energies obtained from 5-fold cross-validation.

Supplementary Tables

Supplementary Table 1: Comparison of adsorption energy prediction studies on solid surfaces using different features and models.

Type	Feature(s)	Model	Data Size and Composition	Accuracy	Ref.
Geometric	Generalized coordination number	Linear regression	~20; OH on Pt surfaces	MAE: 0.056 eV	[1]
Geometric	Orbital wise coordination number	Linear regression	~30; CO on Au surfaces<br>~40; CO, O on Au core shell alloys	RMSE: 0.06-0.19 eV	[2]
Geometric	Adjusted coordination number	Linear regression	~30; H on Co_3O_4 surfaces	RMSD: 0.169 eV	[3]
Geometric	Atomic distances and connectivity	Random forest regression	~2000; CO on $Au_x(SR)_y$ nanoclusters	RMSE:0.17 eV	[4]
Geometric	Atomic connectivity graphs + atomic properties	Convolutional neural network	~12000; CO,<br>~12000; H on pure, binary, ternary, quaternary alloys	MAE: 0.13-0.19 eV	[5,6]
Geometric	Atomic positions + symmetry functions	Neural network	~1400; CO, HOCO on Au surfaces	RMSE: 0.05-0.06 eV	[7]
Geometric	Neighbor populations	Linear regression	~1900; O, OH on high entropy Ru, Ir, Rh, Pt, Pd alloys	RMSD: 0.063-0.076 eV	[8]
Electronic	d-band properties + electronegativity	Neural network	~250; CO on bimetallic alloys	RMSE:0.12 eV	[9]
Electronic	LMTO d-band center + electronegativity	Kernel ridge regression	~260; CO on bimetallic alloys	RMSE: 0.08 eV	[10]
Electronic	Atomic properties + electronic + d-band, sp-band properties	Compressed sensing	~900; C, O, H containing adsorbates on single atom and bimetallic alloys	RMSE: 0.15 eV	[11]
Electronic	Atomic radius + s,p,d states near Fermi level	Compressed sensing	~30; CH_4 on M/Cu alloys (M=d-block metals)	MAE: 0.023 eV	[12]
Electronic	Composition, atomic properties, orbital moments	Gaussian process regression	~4000; O, OH on double perovskites	RMSE: 0.40-0.46 eV	[13]
Electronic	O 2p-band center	Linear regression	~900; O, OH on oxygen in metals, rutile/perovskite oxides	MAE: 0.23-0.37 eV	[14]
**Electronic**	**Electronic density of dtates**	**Convolutional neural network**	**~37000; H, C, N, O, S, CH, CH_2, CH_3, NH, OH, SH**	**MAE: 0.116 eV**	**This work**
Energetic	Monatomic adsorption energy	Linear regression	~160; AH_x (A=C, N, O, S), on late d-block metals	MAE: 0.13 eV	[15]
Energetic	Monatomic adsorption energy	Linear regression	~80; OH, NH, NH_2, SH on oxides, nitrides, sulfides	MAE: ~0.19 eV	[16]

Energetic	Vacancy formation energy	Linear regression	~40; H adsorption on doped Co_3O_4 surfaces	MAE: 0.16 eV	[17]
Energetic	Bulk, vacancy formation energy, atomic properties	Compressed sensing	~90; single metal atoms on metal oxides	RMSE: ~0.4-0.5 eV	[18]
Energetic	Monatomic adsorption energies and coordination numbers	Linear regression	~40; OH, CH, CO, CH_3, on bimetallic alloys	MAE: 0.09-0.18 eV	[19]
Energetic	Adsorption energy of O, OH, CCHOH	Principal component regression	~31000; 71 molecular fragments from C1 and C2 alcohols on transition metals	MAE: 0.12-0.19 eV	[20]
Energetic	Adsorption energies of various species	Gaussian process regression	~37000; H, C, N, O, S, CH, CH_2, CH_3, NH, OH, SH	RMSE: 0.09-0.27 eV	[21]

Supplementary References

1. Calle-Vallejo F., Martínez J. I., García-Lastra J. M., Sautet P., Loffreda D. Fast Prediction of Adsorption Properties for Platinum Nanocatalysts with Generalized Coordination Numbers. *Angew. Chem. Int. Ed.* **53,** 8316-8319 (2014).
2. Ma X., Xin H. Orbitalwise Coordination Number for Predicting Adsorption Properties of Metal Nanocatalysts. *Phys. Rev. Lett.* **118,** 036101 (2017).
3. Fung V., Tao F. F., Jiang D. E. General Structure-Reactivity Relationship for Oxygen on Transition-Metal Oxides. *J. Phys. Chem. Lett.* **8,** 2206-2211 (2017).
4. Panapitiya G., Avendaño-Franco G., Ren P., Wen X., Li Y., Lewis J. P. Machine-Learning Prediction of CO Adsorption in Thiolated, Ag-Alloyed Au Nanoclusters. *J. Am. Chem. Soc.* **140,** 17508-17514 (2018).
5. Back S., Yoon J., Tian N., Zhong W., Tran K., Ulissi Z. W. Convolutional Neural Network of Atomic Surface Structures To Predict Binding Energies for High-Throughput Screening of Catalysts. *J. Phys. Chem. Lett.* **10,** 4401-4408 (2019).
6. Gu G. H., Noh J., Kim S., Back S., Ulissi Z., Jung Y. Practical Deep-Learning Representation for Fast Heterogeneous Catalyst Screening. *J. Phys. Chem. Lett.* **11,** 3185-3191 (2020).
7. Chen Y., Huang Y., Cheng T., Goddard W. A. Identifying Active Sites for CO2 Reduction on Dealloyed Gold Surfaces by Combining Machine Learning with Multiscale Simulations. *J. Am. Chem. Soc.* **141,** 11651-11657 (2019).
8. Batchelor T. A. A., Pedersen J. K., Winther S. H., Castelli I. E., Jacobsen K. W., Rossmeisl J. High-Entropy Alloys as a Discovery Platform for Electrocatalysis. *Joule* **3,** 834-845 (2019).
9. Ma X., Li Z., Achenie L. E. K., Xin H. Machine-Learning-Augmented Chemisorption Model for CO2 Electroreduction Catalyst Screening. *J. Phys. Chem. Lett.* **6,** 3528-3533 (2015).
10. Noh J., Back S., Kim J., Jung Y. Active learning with non-ab initio input features toward efficient CO2 reduction catalysts. *Chemical Science* **9,** 5152-5159 (2018).
11. Andersen M., Levchenko S. V., Scheffler M., Reuter K. Beyond Scaling Relations for the Description of Catalytic Materials. *ACS Catal.* **9,** 2752-2759 (2019).
12. Fung V., Hu G., Sumpter B. Electronic band contraction induced low temperature methane activation on metal alloys. *J. Mater. Chem. A* **8,** 6057-6066 (2020).
13. Li Z., Achenie L. E. K., Xin H. An Adaptive Machine Learning Strategy for Accelerating Discovery of Perovskite Electrocatalysts. *ACS Catal.* **10,** 4377-4384 (2020).
14. Dickens C. F., Montoya J. H., Kulkarni A. R., Bajdich M., Nørskov J. K. An electronic structure descriptor for oxygen reactivity at metal and metal-oxide surfaces. *Surf. Sci.* **681,** 122-129 (2019).
15. Abild-Pedersen F., Greeley J., Studt F., Rossmeisl J., Munter T., Moses P. G.*, et al.* Scaling Properties Of Adsorption Energies For Hydrogen-Containing Molecules On Transition-Metal Surfaces. *Phys. Rev. Lett.* **99,** 016105 (2007).
16. Fernández E. M., Moses P. G., Toftelund A., Hansen H. A., Martínez J. I., Abild-Pedersen F.*, et al.* Scaling relationships for adsorption energies on transition metal oxide, sulfide, and nitride surfaces. *Angew. Chem.* **120,** 4761-4764 (2008).
17. Fung V., Tao F. F., Jiang D.-e. Trends of Alkane Activation on Doped Cobalt (II, III) Oxide from First Principles. *ChemCatChem* **10,** 244-249 (2018).
18. O'Connor N. J., Jonayat A. S. M., Janik M. J., Senftle T. P. Interaction trends between single metal atoms and oxide supports identified with density functional theory and statistical learning. *Nat. Catal.* **1,** 531-539 (2018).

19. Choksi T. S., Roling L. T., Streibel V., Abild-Pedersen F. Predicting Adsorption Properties of Catalytic Descriptors on Bimetallic Nanoalloys with Site-Specific Precision. *J. Phys. Chem. Lett.* **10,** 1852-1859 (2019).
20. García-Muelas R., López N. Statistical learning goes beyond the d-band model providing the thermochemistry of adsorbates on transition metals. *Nat. Commun.* **10,** 4687 (2019).
21. Osman M., Kirsten W., Jacob B., Thomas B. A Bayesian Framework for Adsorption Energy Prediction on Bimetallic Alloy Catalysts. *10.26434/chemrxiv.10295129.v1* (2019).
